# Overexpression of ultraconserved region 83- induces lung cancer tumorigenesis

**DOI:** 10.1371/journal.pone.0261464

**Published:** 2022-01-11

**Authors:** Ivan Vannini, Manuela Ferracin, Francesco Fabbri, Muller Fabbri

**Affiliations:** 1 Biosciences Laboratory, IRCCS Istituto Romagnolo per lo Studio dei Tumori (IRST) "Dino Amadori", Meldola, Italy; 2 Department of Experimental, Diagnostic and Specialty Medicine—DIMES, University of Bologna, Bologna, Italy; 3 Center for Cancer and Immunology Research, Children’s National Hospital, Washington, DC, United States of America; University of Nebraska Medical Center, UNITED STATES

## Abstract

The expression of non–coding RNAs (ncRNAs) is dysregulated in human cancers. The transcribed ultraconserved regions (T-UCRs) express long ncRNAs involved in human carcinogenesis. T-UCRs are non-coding genomic sequence that are 100% conserved across humans, rats and mice. Conservation of genomic sequences across species intrinsically implies an essential functional role and so we considered the expression of T-UCRs in lung cancer. Using a custom microarray we analyzed the global expression of T-UCRs. Among these T-UCRs, the greatest variation was observed for antisense ultraconserved element 83 (*uc*.*83-*), which was upregulated in human lung cancer tissues compared with adjacent non cancerous tissues. Even though *uc*.*83-* is located within the long intergenic non-protein coding RNA 1876 (*LINC01876*) gene, we found that the transcribed *uc*.*83-* is expressed independently of *LINC01876* and was cloned as a 1143-bp RNA gene. In this study, functional analysis confirmed important effects of *uc*.*83-* on genes involved in cell growth of human cells. siRNA against *uc*.*83-* decreased the growth of lung cancer cells while the upregulation through a vector overexpressing the *uc*.*83-* RNA increased cell proliferation. We also show the oncogenic function of *uc*.*83-* is mediated by the phosphorylation of AKT and ERK 1/2, two important biomarkers of lung cancer cell proliferation. Based on our findings, inhibition against *uc*.*83-* could be a future therapeutic treatment for NSCLC to achieve simultaneous blockade of pathways involved in lung carcinogenesis.

## Introduction

Non-small-cell lung cancer (NSCLC) is the prevalent form of lung cancer with a 5-year survival of ~15% [1]. Although improvements in treatment alternatives such as radiation, surgery, chemotherapy, targeted therapy and immunotherapy, prognosis is poor. Dysregulation of non-coding RNAs (ncRNAs) causes cancer. Increasing evidence suggests an important regulatory function of long ncRNAs in cellular processes [2–5]. Conserved sequences of ncRNAs across species may indicate a cellular role. A genome-wide research identified 481 genomic sequences longer than 200 bp that demonstrated a conservation with 100% identity across rat, mouse and human genomes. These ultraconserved regions (UCRs) were proved to be transcribed as ultraconserved ncRNAs (T-UCRs) [6]. T-UCRs show different profiles in various human cancers and regulate cellular proliferation and apoptosis. Dysregulation of T-UCRs is observed in several types of hematological and solid tumors, compared to the normal tissue counterpart and are often situated at cancer-associated genomic regions [7–10]. It was demonstrated (including contributions by our group) that *uc*.*73A* is a oncogene T-UCR in colorectal cancer (CRC) cell lines and other groups showed *uc*.*338* and *uc*.*73A* as oncogenes in hepatocellular carcinoma (HCC) and CRC, respectively [11, 12]. Also, other groups showed that Single Nucleotide Polymorphisms (SNPs) in T-UCR genes are associated with familial breast cancer risk [13]. We have previously shown that T-UCRs are aberrantly expressed in lung cancer and in particular we discovered that transcribed *uc*.*339* is overexpressed in NSCLC cells causing an increase of cell proliferation [14]. Also, we recently contributed to the discovery that *uc*.*8* causes bladder carcinogenesis by binding with *miR-596* [15]. Together, these results confirm a function of T-UCRs in human carcinogenesis. In this study we observed that the most differentially expressed T-UCR in NSCLC primary tumors compared to the non-cancerous lung tissue was *uc*.*83-*. We evaluated the oncogenic function of *uc*.*83-* in lung cancer cells and its regulation of downstream target proteins involved in tumor cell proliferation. Our findings confirm an oncogenic function for *uc*.*83-* in lung cancer.

## Materials and methods

### Patient samples and cell lines

Paired frozen cancerous tissue and adjacent normal lung from 18 patients diagnosed with NSCLC were provided by the IRCCS Istituto Romagnolo per lo Studio dei Tumori (IRST) "Dino Amadori", in Meldola (FC), Italy. All subjects gave their informed consent for inclusion before they participated in the study. The study was conducted in accordance with the Declaration of Helsinki, and the protocol was approved by local ethics committee (CEROM, IRSTB126). Lung cancer cell lines were obtained from American Type Culture Collection and were cultured as a monolayer at 37°C. H1299 (lung adenocarcinoma), H358 (bronchioalveolar carcinoma) and H460 (large cell lung cancer) cells were kept in RPMI 1640 (ATCC) with 10% FBS while A549 (lung adenocarcinoma) cells were maintained in F12K medium (ATCC), supplemented with 10% FBS. Mycoplasma Analysis was performed every two months on all cell lines (MycoAlert™ Mycoplasma Detection Kit–Lonza).

### RT-qPCR

Total RNA extraction was performed using TRIzol® reagent (Invitrogen) and DNA was eliminated with TURBO DNA-free™ kit (Ambion). To test RNA quality and concentration, NanoDrop® ND-1000 Spectrophotometer (Thermo Scientific) was used. *uc*.*83-* was retrotranscripted using the RT *uc*.*83-* forward primer (5’-CTTGGCCAGCTTTCATCCTC-3’) and the TaqMan® MicroRNA Reverse Transcription kit (Applied Biosystems). Pre-amplification of the *uc*.*83-* cDNA was tested with TaqMan® PreAmp Master Mix (Applied Biosystems). Next, RT-qPCR was performed in triplicate with TaqMan® Universal PCR Master Mix (Applied Biosystems) as indicated by the company’s manual. Normalization of RT-qPCR was obtained with RNU44. To achieve RT-qPCR reactions, Applied Biosystems® 7500 Real-Time PCR System (Applied Biosystems) was used.

### Rapid Amplification of cDNA ends (RACE)

To detect the 5′- and 3′-end of the *uc*.*83-* transcript, RNAs obtained from silencing *LINC01876* H358 and H1299 cells were treated with DNase I (RNase-free) (Invitrogen) and the SMARTer RACE cDNA Amplification Kit (Clontech) was used, following the operator’s instructions. Amplification of the cDNA ends were obtained with the Platinum Taq DNA Polymerase High Fidelity (Invitrogen) and gene-specific primers were used as follows: 5’-CTTGGCCAGCTTTCATCCTC*-*3’ for *uc*.*83-* 5’ RACE and 5’-AGCAATATTGGTGCAGGAGGT-3’ for *uc*.*83-* 3’ RACE. Furthermore, we carried out a nested PCR with the provided nested universal primer and the nested gene primers as follows: 5’-GGTGACAGTCTAGCGTCAAG-3’ for *uc*.*83-* Nested 5’ RACE and 5’-CCATTGCTTAGGAAAATTGAGCTTT-3’ for *uc*.*83-* Nested 3’ RACE. Reaction controls were Placental RNA and Transferrin receptor-specific primers. The PCR sequences were then analyzed on a 1.5% agarose gel, and DNA extraction was performed using the QIAquick Gel Extraction Kit (Qiagen). To sequence the RACE fragments, the T7 and T3 primers were used. Next, the RACE sequences were blasted with the UCSC Genome Browser website (http://genome.ucsc.edu/cgi-bin/hgBlat?command=start).

### Reagents and transfection conditions

The cloning of *uc*.*83-* RACE sequence into the pCDH-CMV-MCS-EF1- copGFP expression vector (pCDH *uc*.*83-*) (System Biosciences) was obtained with the following specific primers as follows: 5’-GCGAATTCACAGATGCTTTTGTCCACAG-3’ for cloning *uc*.*83-* with EcoRI restriction site and 5’-GCGGATCCTTTTTGCTTTTAACTGAAAA-3’ for cloning *uc*.*83-* with BamHI restriction site. The transfection of the pCDH *uc*.*83-* plasmid and empty plasmid was performed using Lipofectamine® LTX (Invitrogen), at a concentration of 1 μg ml^−1^. The silencing of the *uc*.*83-* transcript was obtained with two different siRNAs designed as follows: *si uc*.*83- (1)* 5′-AAGAAUGAAAUUUCUAUUGAT -3′ and *si uc*.*83- (2)* 5′-UAAACUGUGAGUUGACACCTG -3′. Ambion provided these two siRNAs and a scrambled siRNA (*si SCR*). The transfection of siRNAs into cell lines at a final concentration of 50 nM was carried out by using Lipofectamine® RNAi Max (Invitrogen).

### Microarray profile and analysis

Total RNA extraction was carried out using TRIzol® reagent (Invitrogen), following the operator’s instructions. To determine the expression of T-UCRs in primary NSCLC tumor tissues and the adjacent normal lung we performed a microarray analysis with custom probes for UCR transcripts. Array hybridization, the T-UCR microarray assembly and target preparation were carried out as described in detail by Sciamanna et al. [16]. The GeneSpring GX software (Agilent Technologies) was used to analyze Microarray results. To set all the negative raw values at 1.0, Data transformation was performed, followed by a log2 transformation and a Quantile normalization. Differentially UCRs expression were observed comparing lung cancer vs. normal samples using a moderated t-test, with 1.5 fold-change filter and Benjamini-Hochberg correction (p-value <0.05). Analyzed Data are showed in S1 Data. The raw data has been deposited on the Array Express public repository with the accession number E-MTAB-11010.

### Western blotting

To analyze the protein expression, immunoblotting experiments were performed on the lysed cells with complete RIPA buffer (Santa Cruz Biotechnologies). Denaturation of cellular proteins was obtained at 100°C for 10 min. Furthermore, Criterion™ XT 4–20% Precast Gels (Bio-Rad) were loaded with 50 μg of proteins. Trans-Blot®Turbo™ Transfer System (Bio-Rad) transferred the proteins on Trans-Blot®Turbo™ Midi Nitrocellulose Transfer Pack membrane (Bio-Rad). Ponceau S (Sigma-Aldrich) membrane staining was used to control the equal amounts of proteins in each lane. Incubation of the membrane for 2 h at room temperature with T-PBS contained 5% non-fat dry milk was performed. The membrane was incubated overnight at 4°C with the primary antibody. Next, horseradish peroxidase conjugated secondary antibody (Dako Corporation) was tested at a dilution of 1:5000. The primary antibodies were: anti- p-ERK1/2 (Thr202 / Tyr204), rabbit polyclonal antibody (Cell Signaling Technology, Cat.#: 9101) diluted 1:1000, anti-ERK1/2, rabbit polyclonal antibody (Cell Signaling Technology, Cat.#: 9102) diluted 1:1000, anti-p-AKT (Ser473), mouse monoclonal antibody (Cell Signaling Technology, Cat.#: 4051) diluted 1:1000, anti-AKT, rabbit polyclonal antibody (Cell Signaling Technology, Cat.#: 9272) diluted 1:1000 and anti-Vinculin, mouse monoclonal antibody (Biohit, Cat.#: 610014) diluted 1:1000. The secondary antibodies were: goat anti-mouse HRP conjugated (Santa Cruz Biotechnology, Cat.#: sc-2005) diluted 1:5000 and goat anti-rabbit HRP conjugated (Bethyl, Cat.#: A120-101P) diluted 1:5000. To observe the bound antibodies, enhanced chemiluminescence and the SuperSignal West Femto Chemiluminescent Substrate (Thermo Scientific) were used. The chemiluminescent bands were quantified by using the Quantity One software (BioRad). All uncropped western blotting images are showed in S1 Raw images.

### Cell-cycle and cell viability assays

For cell-cycle experiment, H1299, H358, H460 and A549 cells were analyzed 72 h after the silencing with *si SCR or si uc*.*83-*, fixed in 70% ethanol, and stained in a solution with 10 μg ml^−1^ of propidium iodide (Sigma-Aldrich), 0.01% of NP40 (Sigma-Aldrich) and 10,000 U ml^−1^ of RNase (Sigma-Aldrich). By flow cytometry using a BD FACSVantage™ cytofluorimeter (BD Biosciences), the samples were observed after incubation of 30–60 min. Data acquisition from 10,000 events for sample was obtained by using the BD CellQuest™ Pro software (BD Biosciences). By using the ModFit LT™ software (Verity Software House), Data were analyzed and showed as percentages of cells in the cell-cycle phases. For cell viability experiment, cells were washed after 72 h from the silencing with *si SCR* / *si uc*.*83-* or the transfection of pCDH *uc*.*83-* / Empty, and resuspended in PBS. The cell suspension was mixed with an identical volume of 0.4% Trypan Blue and incubated for 8–10 min at room temperature. In a KOVA®Glasstic® Slide counting chamber (Hycor Biomedical), total cell numbers and fractions of viable and non-viable stained cells were obtained.

### Statistical analysis of data

Statistical data are shown as mean ± standard deviation (s.d.) of experiments conducted in triplicate. Significance was calculated by two-tailed Student’s t-test. A P value <0.05 was considered statistically significant.

## Results

### *uc*.*83-* expression is increased in NSCLC tumors

**Fig 1:**
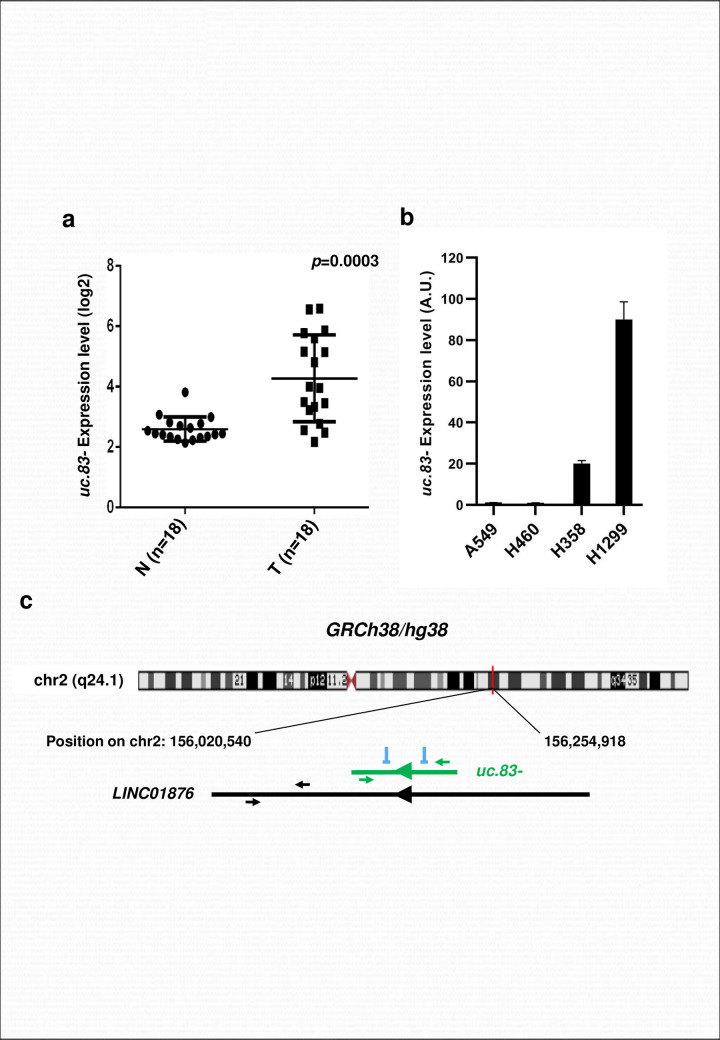
*uc*.*83-* is up-regulated in NSCLC and in cell lines. **(A)** *uc*.*83-* expression in 18 paired primary NSCLC tumor tissues (T) and the adjacent non-cancerous lung (N) obtained from normalized microarray data. Paired t-test P-value < 0.001. **(B)** RT-qPCR for *uc*.*83-* in A549, H460, H358 and H1299 cells. The expression values of *uc*.*83-* were normalized on the RNU44. * P < 0.05. The results are the mean ± s.d. of experiments. **(C)** Chromosomal mapping of *uc*.*83-* locus and of its host gene *LINC01876* as indicated in the GRCh38/ hg38 Human Genome Chromosome. The green arrowheads below and above the bar of the *uc*.*83-* transcript show the position of the primers used for the expression analysis of *uc*.*83-*, while the black arrowheads below and above the bar of the *LINC01876* show the position of the primers used for the expression analysis of *LINC01876* mRNA. The green and black arrowheads inside the bars show the transcriptional direction of the *uc*.*83-* gene and of the *LINC01876* gene, respectively. The symbols ┴ show the sites where the siRNAs against *uc*.*83-* were designed.

We measured UCRs expression profile in 18 paired frozen tumor and adjacent non-tumor lung samples, by microarray analysis. We identified 210 differently expressed UCRs (adjusted p-value < 0.05), as reported in S1 Data. Among the most upregulated T-UCRs in tumors compared to the normal lung tissues we noticed all probes of *uc*.*83-*. We determined the expression of *uc*.*83-* in the tumor and adjacent normal tissues (Fig 1A) and found a statistically significant tumor up-regulation of *uc*.*83-* in cancerous vs non-cancerous adjacent tissues (*P = 0*.*0003*). Also we determined *uc*.*83-* endogenous expression in four human NSCLC cell lines (A549, H460, H358 and H1299) by reverse transcription quantitative PCR (RT-qPCR). The assessed cell lines expressed *uc*.*83-* at different levels, with the highest expression in H358 and H1299 (Fig 1B).

### *uc*.*83-* expression is regulated independently of the Long Intergenic Non-Protein Coding RNA 1876 (*LINC01876*) gene

**Fig 2:**
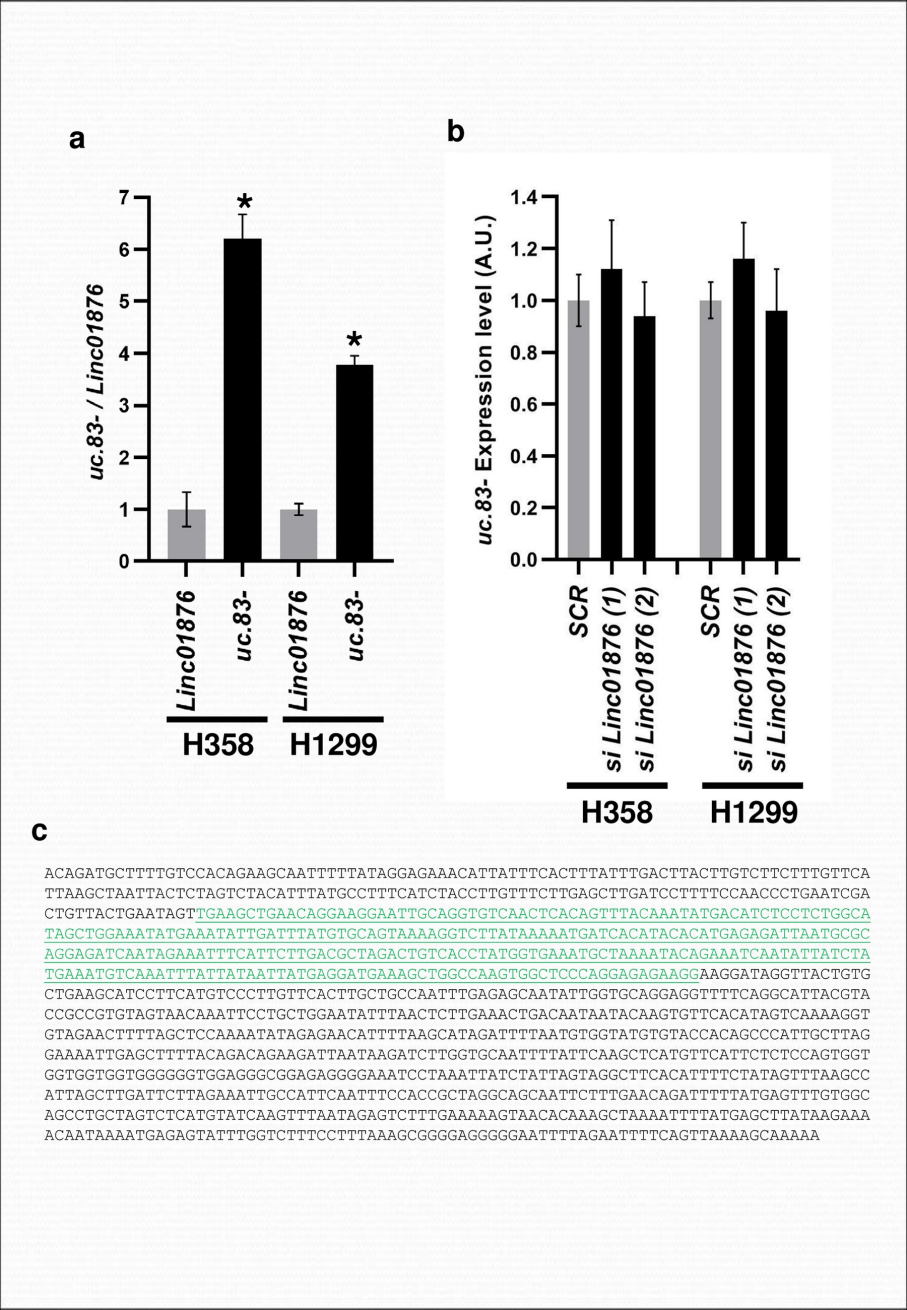
*uc*.*83-* expression is independent of the *LINC01876* gene expression. **(A)** Expression analysis for *uc*.*83-* and *LINC01876* in H358, H1299 cells. The expression values of *uc*.*83-* and *LINC01876* were normalized on the RNU44 and then referred to *LINC01876* expression. * P < 0.05. The results are the mean ± s.d. of experiments. **(B)** Expression analysis for *uc*.*83-* in H358 and H1299 cells transfected with two different *anti-LINC01876* siRNAs (*si LINC01876 (1)* and *(2)*) or an anti-scrambled siRNA (*si SCR*). The expression values of *uc*.*83-* were normalized on the RNU44. * P < 0.05. The results are the mean ± s.d. of experiments. **(C)** Sequence of the *uc*.*83-* transcript, as obtained by RACE. The underlined green bases indicate the sequence as reported by Bejerano et al. [6].

Bejerano et al. (6) observed that *uc*.*83-* consists of 296 nt highly conserved throughout the species. In humans, the *uc*.*83-* ultraconserved region is located entirely within the sequence of the long intergenic non-protein coding RNA 1876 (*LINC01876*) gene on chromosome 2 (Fig 1C). To evaluate the interrelationship between *LINC01876* and *uc*.*83-* transcription, we first examined their expression in lung cancer cell lines. The primers for *uc*.*83-* were designed inside the sequence of this ncRNA while the primers for *LINC01876* were designed outside of the *uc*.*83-* sequence. We performed these experiments in H358 and H1299 cell lines that showed the highest *uc*.*83-* expression. Although *uc*.*83-* is located within the *LINC01876* (Fig 1C), the transcripts of ncRNA encoding *uc*.*83-* and *LINC01876* were expressed differently (Fig 2A). Moreover, *uc*.*83-* expression resulted unchanged in H358 and H1299 cell lines transfected with siRNA against *LINC01876* despite a high reduction in *LINC01876* mRNA expression (Fig 2B and S1 Fig). These data confirm that *uc*.*83-* expression is independent of the *LINC01876* gene expression.

### Identification of the transcript encoding *uc*.*83-*

Since *uc*.*83-* is transcribed independently of *LINC01876*, we cloned the transcript encoding this ultraconserved element. To obtain the 3′ end and the 5′ end of this ncRNA, Rapid amplification of cDNA ends (RACE) was performed. RNAs obtained from silencing *LINC01876* H358 and H1299 cells were reversed transcribed so a complete cDNA with an additional SMARTer sequences at the 5’ and 3’ ends were obtained. The 3′ RACE and 5’ RACE studies identified 179 nt at the 5′ end and 668 nt at the 3’ end upstream and downstream of the ultraconserved sequence detected by Bejerano et al., respectively (6). Therefore, we concluded that the length of the *uc*.*83-* transcript is sequence of 1143 bp (Fig 2C).

### *uc*.*83-* promotes cell growth of NSCLC cell lines

**Fig 3:**
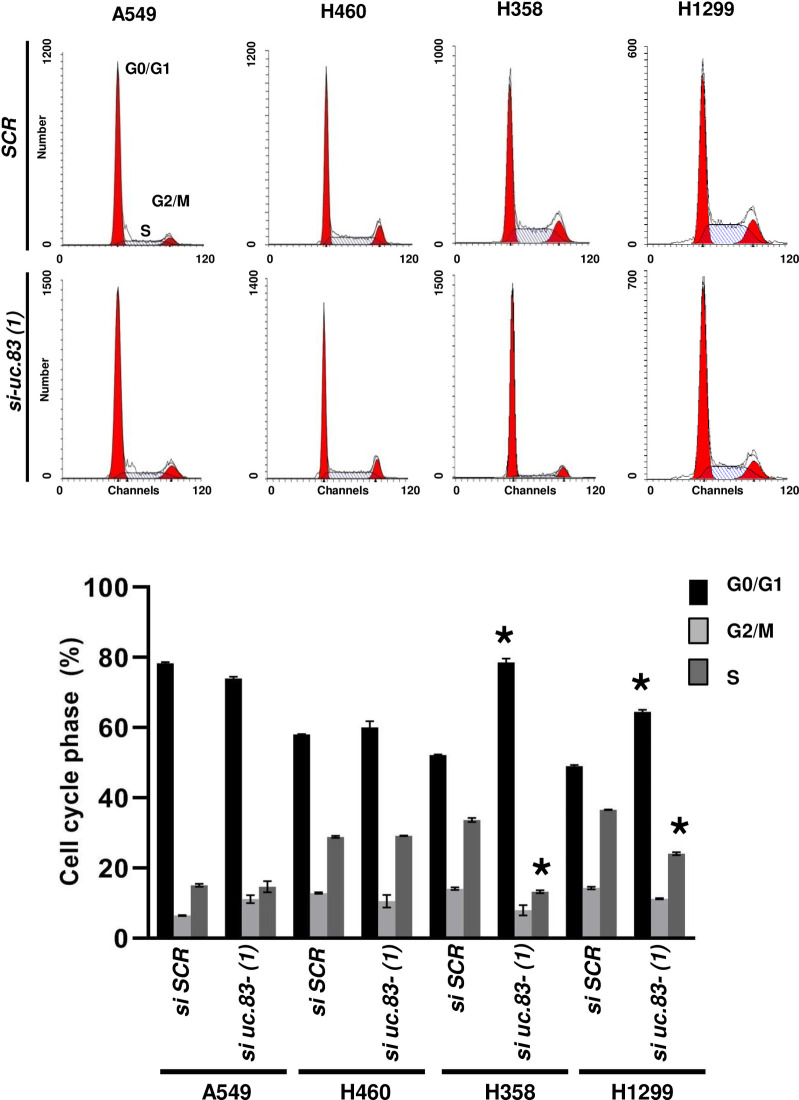
Cell cycle analysis in *uc*.*83-* silencing cells. Cytofluorimetric analysis of Cell cycle with propidium iodide staining in A549, H460, H358 and H1299 cells transfected with *si uc*.*83-(1)* or *si-SCR* for 72h. Cytofluorimetric analysis of hypotonic propidium iodide-stained cells (representative experiments) in the upper part. The percentage of cells in G_0_/G_1_ or G_2_/M or S phase of the cell cycle are represented in the lower part. The results are the mean ± s.d. of experiments. * P<0.05.

**Fig 4:**
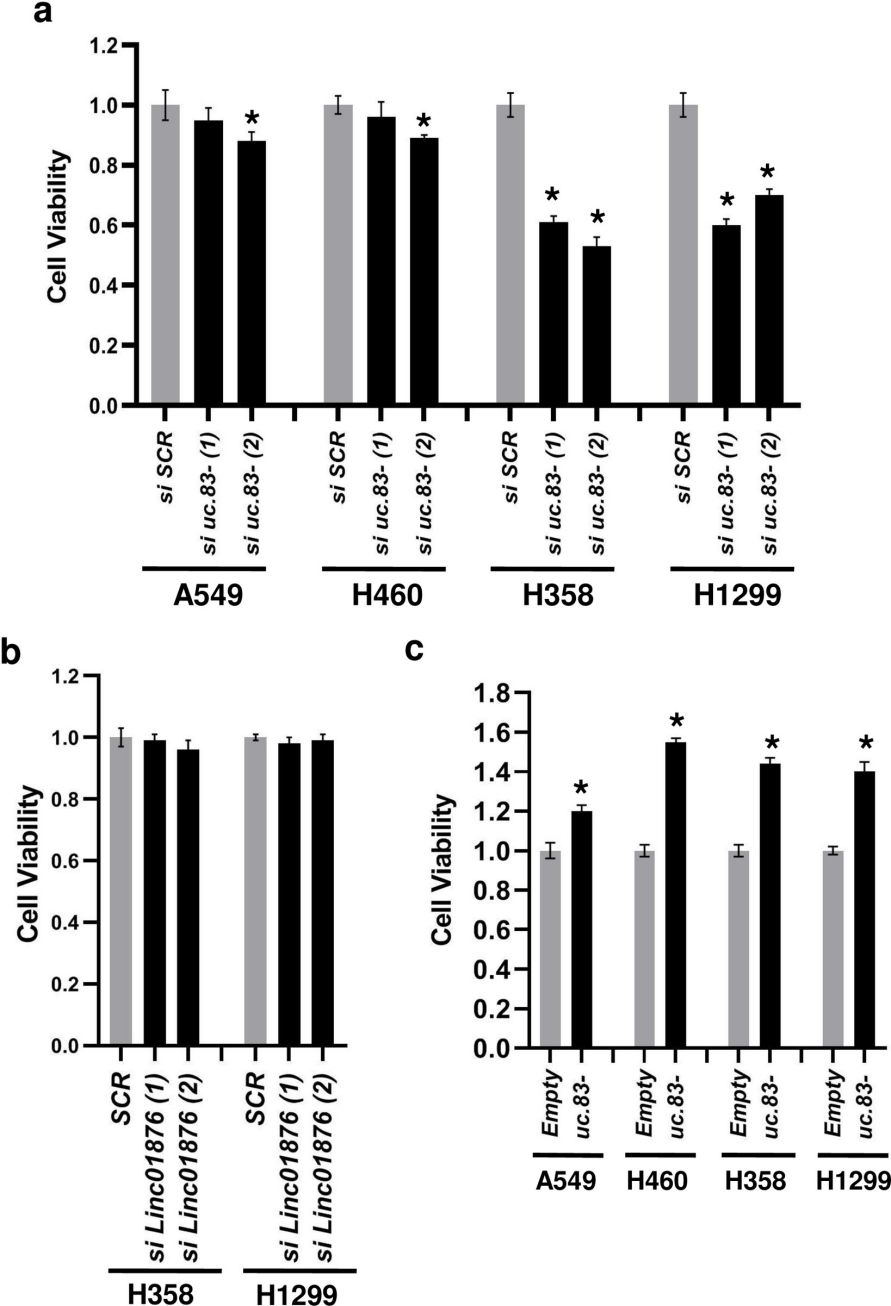
*uc*.*83-* induces cancer cell growth. **(A)** Cell viability assay in A549, H460, H358 and H1299 cells transfected with two different *si uc*.*83-* or *si SCR* for 72 h. The results are the mean ± s.d. of experiments. The represented values were normalized on *si SCR*. * P<0.05. **(B)** Cell viability assay in H358, H1299 cells transfected with two different *si LINC01876* or *si SCR* for 72 h. The results are the mean ± s.d. of experiments. The represented values were normalized on *si SCR*.* P<0.05. **(C)** Cell viability assay in A549, H460, H358 and H1299 cells transfected with a vector overexpressing *uc*.*83-* (*uc*.*83-*) or empty vector (*Empty*) and detected after 72h. The results are the mean ± s.d. of experiments. The represented values were normalized on *Empty*. * P<0.05.

Since *uc*.*83-* transcript in NSCLC primary tissues was expressed at higher levels than the adjacent non-cancerous lung tissues, we tested the functional involvements of *uc*.*83-* expression modulation in lung carcinogenesis. First, we performed a silencing of *uc*.*83-* endogenous expression through two siRNAs against two different regions of the *uc*.*83-* RNA [*si uc*.*83-(1)* and *si uc*.*83-(2)*] in A549, H460, H358 and H1299 cells. Through a growth curve over a course of seven days, we observed that in H358 and H1299 cells (but not in A549 and H460 cells) the growth was significantly reduced starting at 72 h (S2 Fig). By cytofluorimetric analysis, we noted that a reduced expression of *uc*.*83-* increased the fraction of H358 and H1299 cells in G_0_/G_1_ phase and decreased the fraction of cells in S-phase, compared to a siRNA scrambled control (Fig 3 and S3 Fig). We observed little to no variation of cell cycle in A549 and H460 cells which express the lowest endogenous levels of *uc*.*83-* (Fig 1B). Next, we analyzed cell viability after *uc*.*83-* silencing. We discovered a significant decrease of cell viability at 72 h in H358, H1299 (Fig 4A). This result was determined by *uc*.*83-* and not *LINC01876* since no change in cell viability of H358 and H1299 cells was observed upon *LINC01876* silencing (Fig 4B). Also, we observed little to no effect after *uc*.*83-* silencing in A549 and H460 (Fig 4A). Conversely, when we transfected A549, H460, H358 and H1299 cells with a vector overexpressing the 1143 nt *uc*.*83-* RNA (*uc*.*83-*) or empty vector (Empty), we found that *uc*.*83-* transfected cells had a significantly increased viability compared to Empty transfected cells (Fig 4C).

### Expression analysis of *uc*.*83-* regulated genes

**Fig 5:**
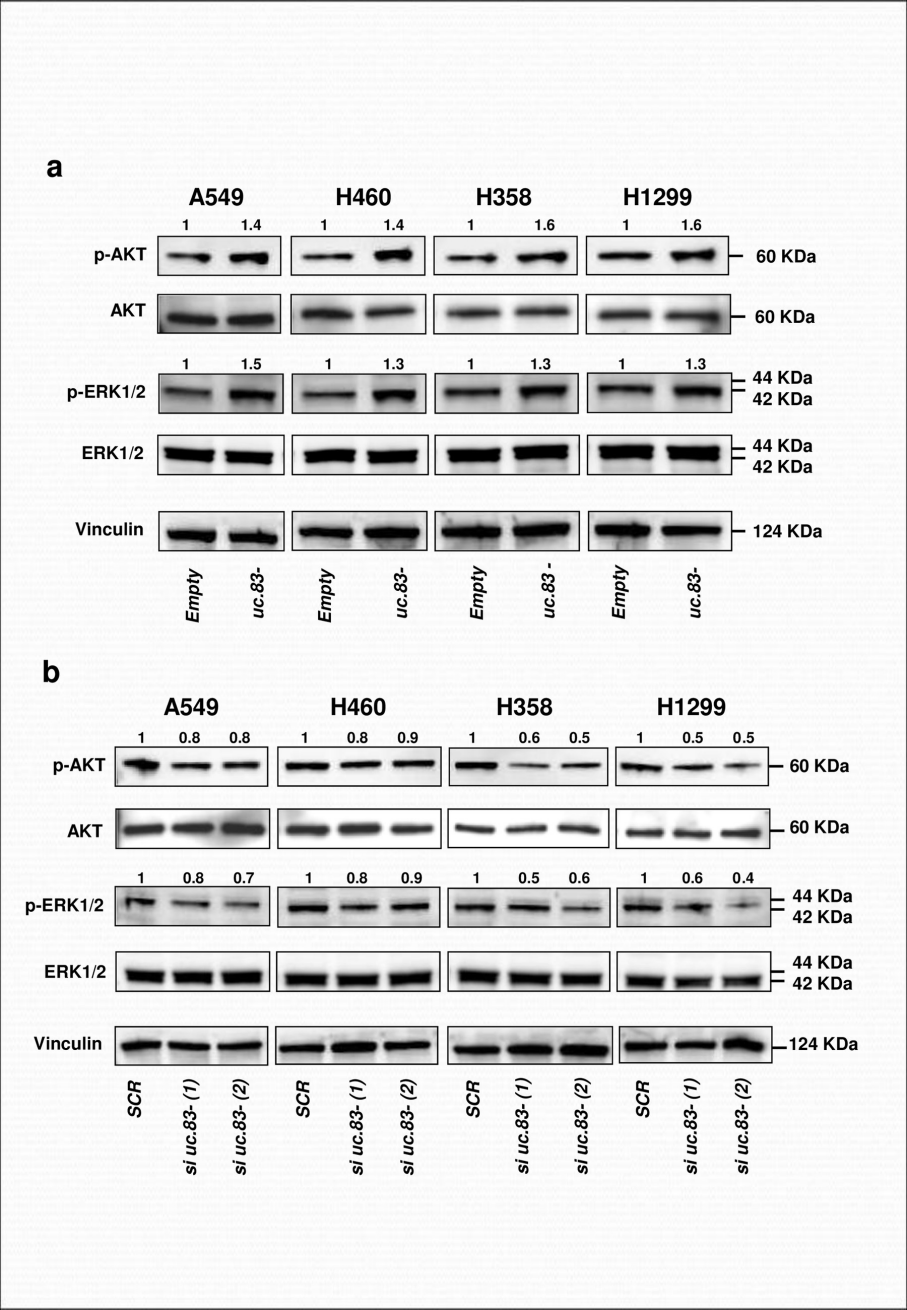
*uc*.*83-* increases the expression of p-ERK1/2 and p-AKT. **(A)** Immunoblotting results for p-ERK1/2, ERK1/2, p-AKT, AKT and Vinculin in A549, H358, H460 and H1299 cells transfected with *uc*.*83-* or Empty vector for 72h. (**B**) Immunoblotting results for p-ERK1/2, ERK1/2, p-AKT, AKT and Vinculin in A549, H460, H358 and H1299 cells transfected with *si uc*.*83-(1)*, *si uc*.*83-(2)* or *si SCR* for 72h. The quantification of the band intensity analyzed with Quantity One software are indicated by the number above the bands. The values ​​were first normalized on Vinculin and *Empty*, then on AKT and ERK 1/2.

Next, we focused on the downstream proteins that were dysregulated by *uc*.*83-* modulation. We decided to focus on Phosphorylated Ser473 protein kinase (p-Akt) and Phosphorylated Thr202/Tyr204 extracellular signal-regulated protein kinases 1/2 (ERK1/2) as they are among the most commonly dysregulated proteins with oncogenic role in NSCLC [17, 18]. In A549, H460, H358 and H1299 cells transfected with a plasmid expressing wild-type *uc*.*83-* we observed an upregulation of p-AKT and p-ERK1/2 protein expressions (Fig 5A). Conversely, silencing of *uc*.*83-* decreased p-AKT and p-ERK1/2 expression in H358 and H1299 (Fig 5B). A modest reduction of the same protein expressions were observed in A549 and H460, likely because of their low *uc*.*83-* endogenous expression (Figs 1B and 5B). Collectively, these results confirm that *uc*.*83-* modulates the expression of p-AKT and p-ERK1/2, determining the activation of the involved pathways and the following cell proliferation.

## Discussion

T-UCRs were described several years ago as highly conserved sequences between species, a condition suggestive of functional roles. Researchers in the field have analyzed the implication of T-UCRs in evolution [19, 20]. We have contributed to some of the seminal discoveries showing the involvement of T-UCRs in human carcinogenesis [12, 14, 15]. We have extended these studies to show aberrantly expressed T-UCRs in lung cancer. Our data confirm a prominent role of T-UCRs in lung cancer cell growth and indicate these RNAs as an uncharacterized group of genes that are implicated in lung carcinogenesis. Our studies identified a ncRNA, *uc*.*83-* as highly expressed in NSCLC primary samples vs adjacent non-cancerous tissue, and whose chromosomal location overlaps with that of *LINC01876*, a long intergenic non-protein coding RNA. We proved that these two genes are independently expressed despite the exonic location of *uc*.*83-* within *LINC01876*. The explanation for the high conservation of *uc*.*83-* across species as rat, mouse, human is unknown. Mobile elements determine genome evolution, and about 50% of our genome derives from characterized transposon DNA [6, 21]. Therefore, it is conceivable that *uc*.*83-* may have originated from exaptation and through an evolutionarily transposable element may have integrated in the genome. Our findings suggest that, *uc*.*83-* exaptation can contribute a cellular role important in cancer cell proliferation. The difference in expression of *uc*.*83-* between normal tissues and lung cancer tissues is noteworthy. Also we demonstrated that *uc*.*83-* increases lung cancer cell growth *in vitro* through a regulation of p-AKT and p-ERK1/2, therefore acting as a *bona fide* oncogene and driver of cancer cell growth. Akt and ERK1/2 activation subsequently causes a number of potential downstream effects. Akt may phosphorylate Mdm2, which downregulates p53-mediated apoptosis [22]. It can also result in inhibition of BAD and BAX, members of proapoptotic Bcl2 family. The nuclear factor kappa-light-chain-enhancer of activated B cells (NFκB) transcription factor is activated by PI3K/AKT pathway and regulates the expression of hundreds of genes which are implicated in cell cycle control, apoptosis, immune modulation, cell adhesion, cell survival and differentiation [23]. Another important gene regulated from Akt activation is the protein kinase, mTOR. It promotes the tumorigenesis, regulation of the cell cycle and inhibition of apoptosis [24]. The serine/threonine ERK1/2 protein kinases are downstream effectors in the MAP kinase cascade, also known as the RAS/RAF/MEK/ERK pathway. Phosphorylated BRAF activates the kinase MEK, which, in turn, activates ERK1/2, resulting in activation of transcriptional factors promoting cell proliferation and cell cycle progression such as Elk1, c-Fos, and c-Jun [25]. These substrates need the nuclear translocation of ERK1/2 for their phosphorylation. Constitutive activation of these AKT and ERK1/2 pathways is a hallmark of cancer and dysregulation of these pathways determinate the initiation, progression and metastastic spread of lung cancer. Targeting of the AKT and ERK1/2 is thus an attractive strategy in the research of novel approaches to treat NSCLC, although single pathway inhibitors have a limited clinical success so far [18]. Also, the inter- and intra-pathway compensatory loops that re-activate the same cascade, either downstream or upstream the point of pharmacological blockade, or activate the different pathway following the blockade of one signaling cascade has been discovered, potentially causing preclinical resistance [26]. Therefore, blocking both pathways might result in a more efficient anti-cancer effect. Inhibition against *uc*.*83-* might be a future therapeutic treatment for lung cancer to reach the blockade of both pathways. Based on our findings supporting a role for *uc*.*83-* in NSCLC, further studies are warranted to discover the mechanistic involvement of *uc*.*83-* in lung carcinogenesis. We are well aware that cancer cells depend on a complex network of transcripts originated from coding and non-coding genes, and that interaction of different RNA classes determines cancer progression more than a single aberration [27–31]. However, this study provides evidence that the aberrant expression of *uc*.*83-* in transformed lung cancer cells promotes NSCLC growth and modulates the AKT and ERK1/2 pathways. The functional role of the *uc*.*83-* transcript in regulating cancer growth may develop the basis for further research on this T-UCR as a new target for NSCLC therapy.

## Supporting information

S1 Fig *LINC01876* expression in *si LINC01876* cell lines. RT-qPCR for *LINC01876* in H358 and H1299 cells transfected with two different *anti-LINC01876* siRNAs (*si LINC01876 (1)* and *(2)*) or an anti-scrambled siRNA (*si SCR*). The expression of *LINC01876* has been normalized to RNU44 and presented as normalized to *si SCR*. * P < 0.05. All data are presented as mean ± s.d. of experiments.
(PPT)

S2 Fig Cell growth analysis in *uc*.*83-* silencing cells. Cell growth assay in A549, H460, H358 and H1299 cells transfected with two different *si uc*.*83-* or *si SCR* at different time. Transfection was repeated at 96 h after the first transfection. Data are presented as mean ± s.d. of experiments. P<0.05.
(PPT)

S3 Fig Cell cycle analysis in *uc*.*83-* silencing cells. Cell cycle analysis (shown as the percentage of cells in G_0_/G_1_ or G_2_/M or S phase of the cell cycle) conducted by cytofluorimetry with propidium iodide staining in A549, H460, H358 and H1299 cells transfected with *si uc*.*83-(2)* or *si SCR* for 72h. Data are presented as mean ± s.d. of experiments. * P<0.05.
(PPT)

S1 Data (XLSX)

S1 Raw images (PDF)
