# Supplementary figures and images for: Overexpression of ultraconserved region 83- induces lung cancer tumorigenesis
Source: PLoS One. 2022 Jan 11;17(1):e0261464. doi: 10.1371/journal.pone.0261464 (PMC8752010; doi:10.1371/journal.pone.0261464)

## Slide 1
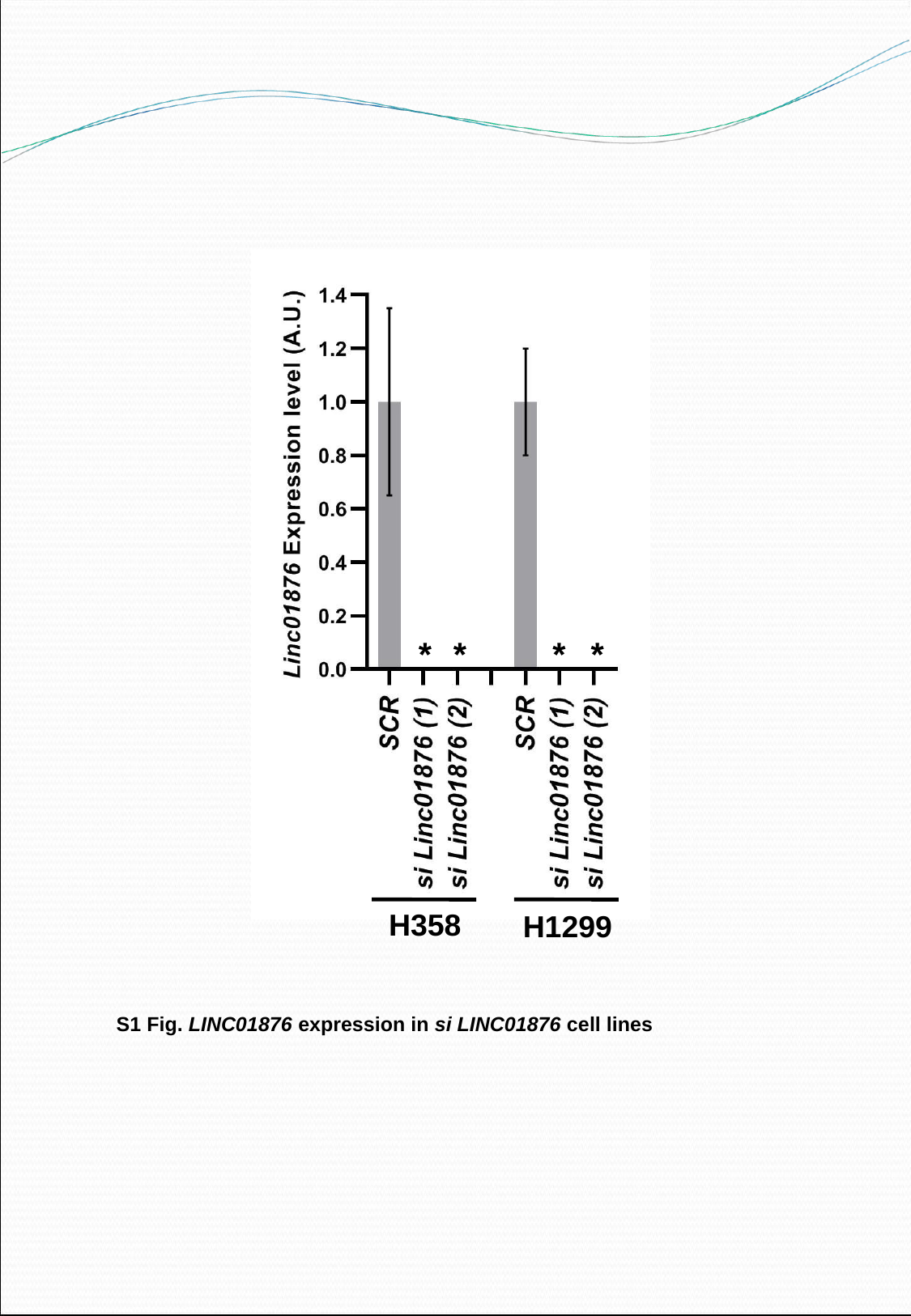

*
*
*
*
 H358
H1299
S1 Fig. LINC01876 expression in si LINC01876 cell lines

Supplement: S1 Fig — RT-qPCR for LINC01876 in H358 and H1299 cells transfected with two different anti-LINC01876 siRNAs (si LINC01876 (1) and (2)) or an anti-scrambled siRNA (si SCR). The expression of LINC01876 has been normalized to RNU44 and presented as normalized to si SCR. * P < 0.05. All data are presented as mean ± s.d. of experiments. (PPT) [file pone.0261464.s001.ppt]

## Slide 1
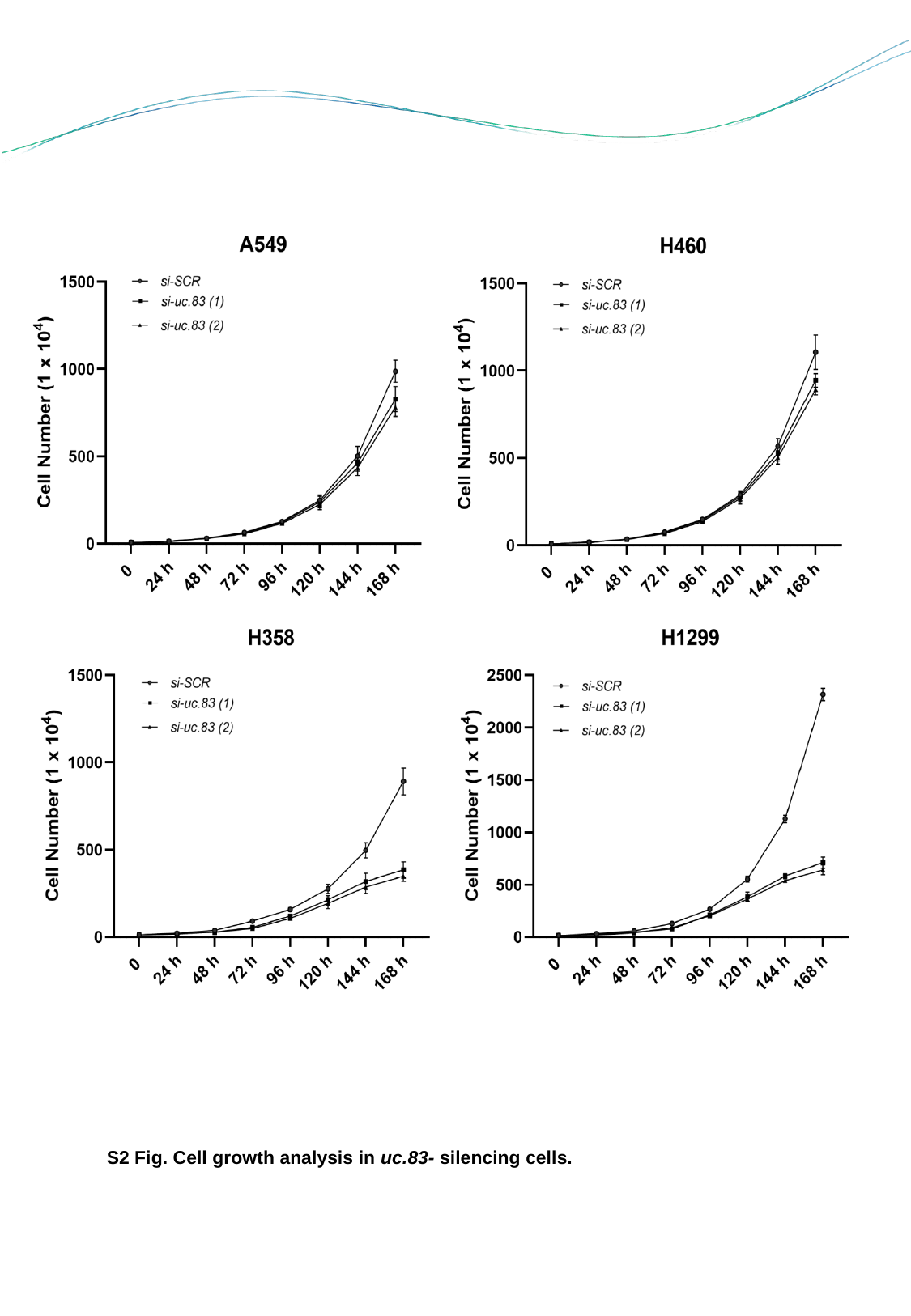

S2 Fig. Cell growth analysis in uc.83- silencing cells.

Supplement: S2 Fig — Cell growth assay in A549, H460, H358 and H1299 cells transfected with two different si uc.83- or si SCR at different time. Transfection was repeated at 96 h after the first transfection. Data are presented as mean ± s.d. of experiments. P<0.05. (PPT) [file pone.0261464.s002.ppt]

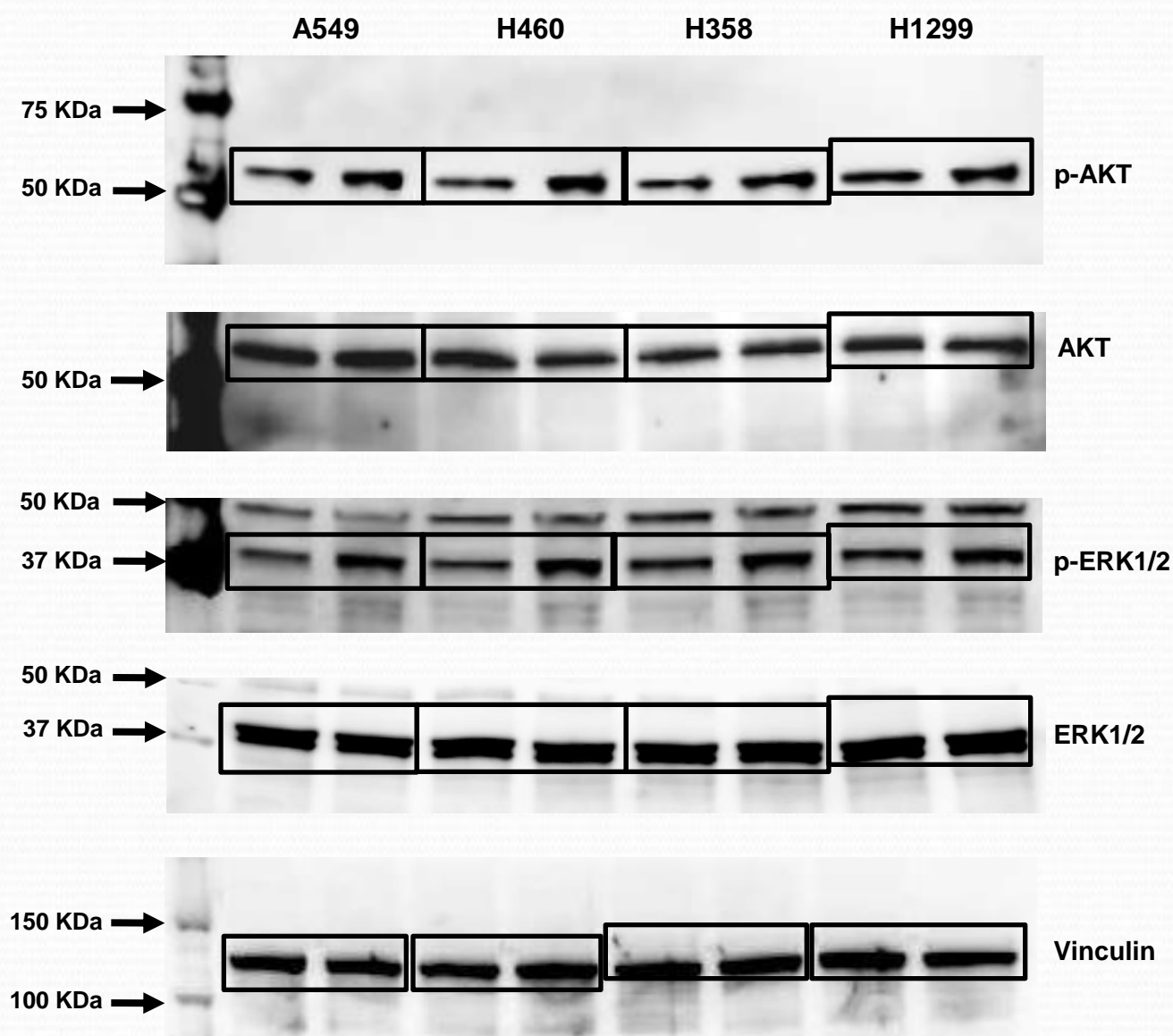

**Uncropped western blotting images for Fig 5a**

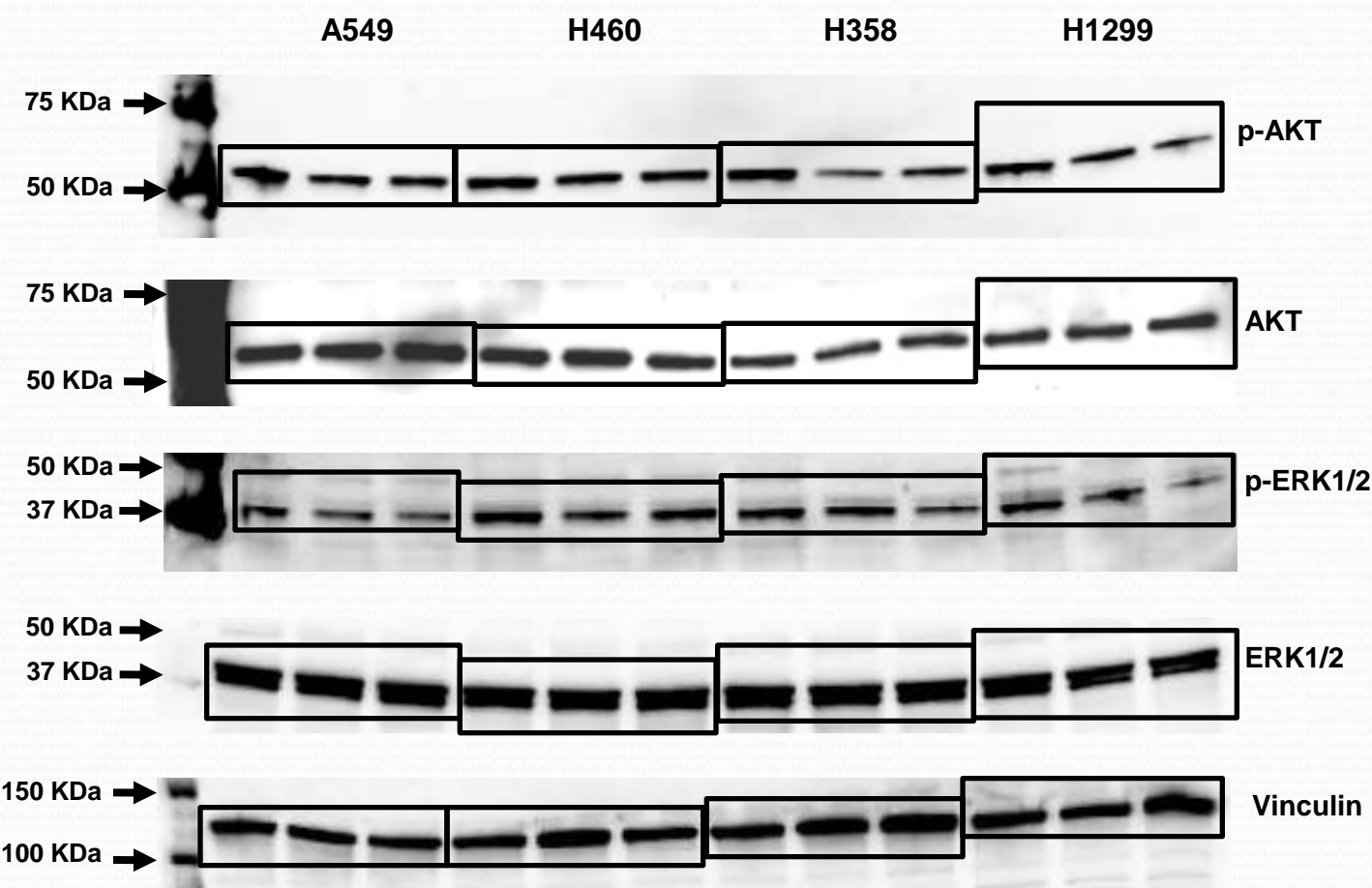

**Uncropped western blotting images for Fig 5b**

Supplement: S1 Raw images — (PDF) [file pone.0261464.s005.pdf]
