# Supplementary material for: Overexpression of ultraconserved region 83- induces lung cancer tumorigenesis
Source: PLoS One. 2022 Jan 11;17(1):e0261464. doi: 10.1371/journal.pone.0261464 (PMC8752010; doi:10.1371/journal.pone.0261464)
Supplement: S3 Fig — Cell cycle analysis (shown as the percentage of cells in G0/G1 or G2/M or S phase of the cell cycle) conducted by cytofluorimetry with propidium iodide staining in A549, H460, H358 and H1299 cells transfected with si uc.83-(2) or si SCR for 72h. Data are presented as mean ± s.d. of experiments. * P<0.05. (PPT) [file pone.0261464.s003.ppt]

## Slide 1
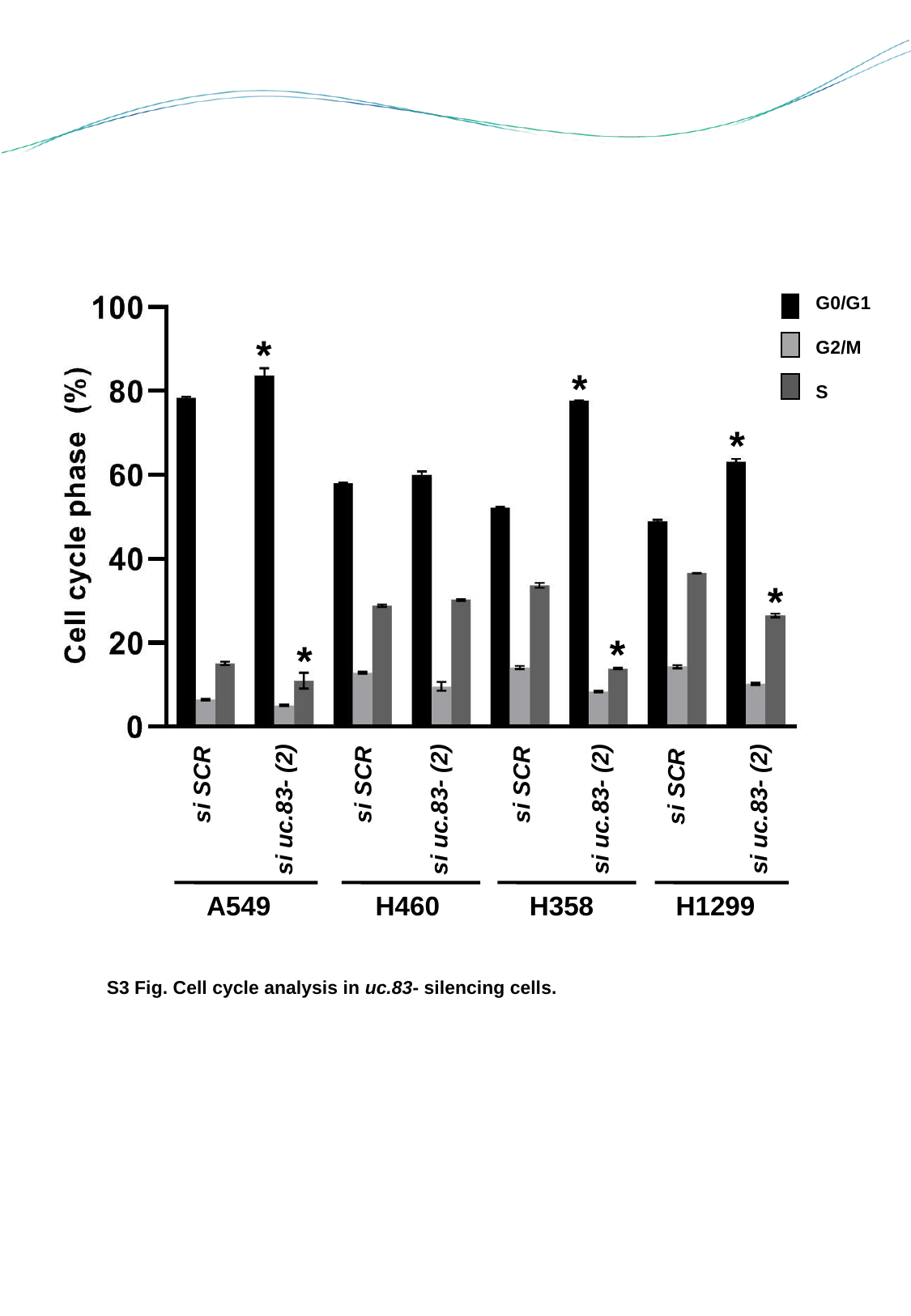

G0/G1
G2/M
S
*
*
*
*
*
*
si SCR
si SCR
si SCR
si SCR
si uc.83- (2)
si uc.83- (2)
si uc.83- (2)
si uc.83- (2)
A549 H460 H358 H1299
S3 Fig. Cell cycle analysis in uc.83- silencing cells.
